# Cardioprotective Potential of *Murraya koenigii* (L.) Spreng. Leaf Extract against Doxorubicin-Induced Cardiotoxicity in Rats

**DOI:** 10.1155/2020/6023737

**Published:** 2020-04-05

**Authors:** Jayasinghe A. N. Sandamali, Ruwani P. Hewawasam, Kamani A. P. W. Jayatilaka, Lakmini K. B. Mudduwa

**Affiliations:** ^1^Department of Medical Laboratory Science, Faculty of Allied Health Sciences, University of Ruhuna, Galle 80000, Sri Lanka; ^2^Department of Biochemistry, Faculty of Medicine, University of Ruhuna, Galle 80000, Sri Lanka; ^3^Department of Pathology, Faculty of Medicine, University of Ruhuna, Galle 80000, Sri Lanka

## Abstract

Dose-dependent cardiotoxicity of doxorubicin may lead to irreversible congestive heart failure. Although multiple mechanisms are involved, generation of free radicals is the most commonly postulated mechanism. Therefore, free radical scavengers are considered as potential therapeutic agents. As *Murraya koenigii* leaves are a rich source of flavonoids and phenols, they have the ability to scavenge free radicals effectively. Therefore, the objective of this study was to investigate the cardioprotective potential of *Murraya* leaf extract against doxorubicin-induced cardiotoxicity in rats. Rats were randomly divided into five groups with 10 animals in each group. Doxorubicin was administered intraperitonially at 18 mg/kg while lyophilized plant extract was administered orally at 2 g/kg. Dexrazoxane, at 180 mg/kg, was used as the positive control. Cardiac damage of doxorubicin control was evident with a significant increase (*p* < 0.05) in cardiac troponin I, NT-pro BNP, AST, and LDH compared to the normal control. Plant-treated group showed cardioprotective effect by significantly reducing (*p* < 0.05) all of the above parameters compared to doxorubicin control (*p* < 0.05). Increased oxidative stress in doxorubicin control was evident with a significant reduction in reduced glutathione, glutathione reductase, glutathione peroxidase, total antioxidant capacity, superoxide dismutase, and catalase activity and a significant increase in lipid peroxidation compared to the control. Interestingly, treatment with *Murraya* leaf extract showed a significant increase in all of the above antioxidant parameters and a significant reduction in lipid peroxidation by showing an antioxidant effect. A significant increase in myeloperoxidase activity confirmed the increased inflammatory activity in doxorubicin control group whereas plant-treated group showed a significant reduction (*p* < 0.05) which expressed the anti-inflammatory effect of *Murraya* leaf extract. Doxorubicin-treated group showed histological evidence of extensive damage to the myocardium while plant-treated group showed a preserved myocardium with lesser degree of damage. Pretreatment with *Murraya* leaf extract may replenish cardiomyocytes with antioxidants and promote the defense against doxorubicin-induced cardiotoxicity.

## 1. Introduction

Anthracyclines are commonly used antineoplastic agents, either alone or in combination with other cytotoxic agents [1]. Among the anthracyclines, doxorubicin and epirubicin are the widely used chemotherapeutic agents in the treatment of cancers including breast, endometrial and gastric, and childhood solid tumours, soft tissue sarcomas, and aggressive lymphoblastic or myeloblastic leukemia. However, the use of anthracyclines is limited by its dose-dependent cardiotoxicity which is evident as impaired left ventricular systolic function or heart failure in 7–26% of the patients treated with 550 mg/m^2^ cumulative dosage of doxorubicin, 0.9–11.4% of the patients treated with 900 mg/m^2^ cumulative dose of epirubicin, and 5–18% of the patients treated with >90 mg/m^2^ cumulative dose of idarubicin [2]. Doxorubicin-induced cardiotoxicity may be manifested as arrhythmias, ischaemia, systolic dysfunction, and heart failure, and the major reasons for these changes are cardiac cell death and necrosis [3].

Multiple mechanisms are involved in doxorubicin-induced cardiotoxicity. Some of them are oxidative stress induced by reactive oxygen species (ROS), topoisomerase II inhibition, and deoxyribonucleic acid (DNA) double strand break leading to transcriptional alteration of the genes and apoptosis, activation of apoptotic pathway by the impairment of mitochondrial function, and intracellular calcium dysregulation [1, 4]. Among these mechanisms, oxidative stress is the most common cause of doxorubicin-induced cardiotoxicity [5]. This occurs as a result of an imbalance between the produced ROS and the intrinsic antioxidant mechanism in the cardiomyocytes [1]. The mitochondrion plays a key role in doxorubicin-induced cardiotoxicity as anionic cardiolipin present in the inner mitochondrial membrane has high affinity to bind with cationic drug doxorubicin [1, 4]. Nicotinamide adenine dinucleotide hydrogen (NADH) dehydrogenase in mitochondria reduces the doxorubicin into semiquinone radical which reacts with molecular oxygen to form superoxide radical which is subsequently responsible for the production of hydrogen peroxide and hydroxyl radical [6]. In addition, endothelial-specific nitric oxide synthase (eNOS) reductase available in the cardiomyocytes also reduces the doxorubicin to form superoxide radicals while transforming eNOS from nitric oxide (NO) to a superoxide producer [1]. Doxorubicin may also directly complex with iron and catalyze the Fenton reaction which converts hydrogen peroxide to hydroxyl radical [7]. Cardiomyocytes are more susceptible than other tissues due to low levels of antioxidants, high reliance on oxidative substrate metabolism, and presence of high volume of mitochondria compared to the tumour cells [6].

*Murraya koenigii* (L.) Spreng. (curry leaf) belonging to the family Rutaceae is native to India, Sri Lanka, and other south Asian countries. The leaves are commonly used in various cuisines as a natural flavouring agent. In traditional ayurvedic medicine, leaves are used to treat diabetes as well as heart diseases, blood disorders, diarrhoea, dysentery, eruption, inflammation, itching, kidney pain, leukoderma, piles, snakebite, thirst, and vomiting [8, 9]. Various *in vitro* and *in vivo* studies have shown that *Murraya koenigii* leaf extract has a vast number of therapeutic applications as it possesses antioxidant, antimutagenic, anti-inflammatory, antibacterial, antifungal, and cytotoxic effects and cardioprotective activities [8, 10–14]. It is widely reported that *Murraya* leaf extract is a rich source of phenolic compounds and alkaloids (carbazole alkaloids) which contribute to the antioxidant activity by scavenging free radicals effectively [8, 9, 15]. As the major mechanism of doxorubicin-induced cardiotoxicity is oxidative stress which is caused by an imbalance between intracellular antioxidant system and the ROS production during doxorubicin metabolism, it is believed that enrichment of heart muscles with antioxidants may be important to prevent cardiotoxicity induced by doxorubicin [16]. Therefore, supplementation therapies with high amounts of antioxidants have been identified as effective measures to prevent doxorubicin-induced cardiotoxicity [17, 18]. As the previous studies have confirmed that *Murraya koenigii* leaf extract has a high antioxidant activity we selected this plant for the present study. Additionally, a study done by Mitra et al. has shown that aqueous *Murraya* leaf extract has a cardioprotective effect against cadmium-induced oxidative stress in rats [19]. As the major mechanism of doxorubicin-induced cardiotoxicity is also the oxidative stress, the objective of this study was to investigate the cardioprotective potential of *Murraya koenigii* (L.) Spreng. aqueous leaf extract against doxorubicin-induced cardiotoxicity in Wistar rats.

## 2. Materials and Methods

### 2.1. Preparation of Plant Extract

The leaves of *Murraya koenigii* (L.) Spreng. were collected from Matara district in the Southern province of Sri Lanka. Botanical identity was determined according to the descriptions given by Jayaweera [20] and confirmed by comparing authentic samples by the curator of the National Herbarium, Royal Botanical Gardens, Peradeniya, Sri Lanka. A voucher specimen (2015/PG/VS/01) was deposited at the Department of Biochemistry, Faculty of Medicine, University of Ruhuna, Sri Lanka.

Plant extracts were prepared according to a method that was optimized and modified previously in our laboratory [21]. The leaves of *Murraya* were dried at 40°C until a constant weight was reached and coarsely grounded. Grounded plant material (24.00 g) was dissolved in 550.0 mL of distilled water and refluxed for 4 hrs. The mixture was strained and the final volume was adjusted to 500.0 mL and freeze-dried.

### 2.2. Experimental Animals

Healthy male and female Wistar albino rats, 6–8 weeks old, weighing 175 ± 25 g, were purchased from the animal house of the Medical Research Institute, Colombo, Sri Lanka, to carry out the experiment. The animals were housed in a well-ventilated animal house at the Faculty of Medicine, University of Ruhuna, Sri Lanka. They were maintained on a standard laboratory diet of rat pellets and water *ad libitum*. Rats were acclimatized to the environment (temperature, 23 ± 2°C; relative humidity, 50 ± 5%; and 12-hour light-dark cycle) for one week prior to experimental use. All protocols used in this study were approved by the Ethical Review Committee of the Faculty of Medicine, University of Ruhuna, Sri Lanka (23.10.2014 : 3.10), guided by the CIOMS international guiding principles of biomedical research involving animals.

### 2.3. Acute and Subchronic Toxicity Evaluation of Plant Extract

The concentration of the plant extract (2.0 g/kg) which gave the highest cardioprotective activity against doxorubicin-induced cardiotoxicity was selected according to a preliminary study conducted for five doses of plant extracts around the human therapeutic dose used in ayurvedic treatment. Acute toxicity study was performed following the Organization for Economic Cooperation and Development (OECD) guideline 423, fixed dose procedure [22]. A group of healthy Wistar albino rats of both sexes (*n* = 10/group) received 2.0 g/kg aqueous extracts of *Murraya koenigii* (L.) Spreng. leaves once orally while the untreated healthy control group received distilled water. Animals were observed individually during the first 30 min, periodically during the first 24 h, with special attention given during the first 4 hours, and daily for a total of 14 days. Observations included changes in skin, fur, eyes, mucous membranes, and behavior pattern. Special attention was directed to the observations of tremors, convulsions, salivation, diarrhea, lethargy, sleep, and coma.

The subchronic toxicity study was performed following the protocol described by the OECD guideline 407 for testing chemicals [23]. Healthy Wistar albino rats were randomly divided into two groups (*n* = 10/group). The first group served as the control group and received distilled water (10 mL/kg) orally for one month. The rats in the second group received the aqueous leaf extract of *Murraya koenigi* (L.) Spreng. (2.0 g/kg) orally for one month. The animals were observed for signs of toxicity and mortality throughout the experimental period. At the end of the experimental period all animals were fasted (16 hrs) and sacrificed by cervical dislocation. Blood samples were collected by cardiac puncture for the assessment of haematological and serum biochemical parameters. The heart, liver, small intestine, spleen, and kidney were carefully isolated, weighed, and fixed in 10% buffered formalin for the histological assessment of tissue damage.

Total white blood cell (WBC) count, total red blood cell (RBC) count, platelet count, hemoglobin concentration, haematocrit (packed cell volume), red blood cell indices including, mean corpuscular volume (MCV), mean corpuscular hemoglobin (MCH), and mean corpuscular hemoglobin concentration (MCHC) were measured as haematological parameters. Serum concentrations of alanine aminotransferase (ALT), aspartate aminotransferase (AST), and alkaline phosphatase (ALP) were estimated using spectrophotometric enzyme assay kits to detect toxic effects of plant extract on liver function, and serum concentrations of creatinine and blood urea were estimated using spectrophotometric enzyme assay kits to detect toxic effects of plant extract on kidney function.

### 2.4. Experimental Procedure for the Cardioprotective Effects of the Plant Extract

The experimental groups were selected according to the method used by Hamza et al. [24]. Dexrazoxane was used as the positive control as it is the only FDA (Food and Drug Administration) approved cardioprotective agent for the treatment of anthracycline-induced cardiotoxicity [25]. Dose of plant extract was selected according to the preliminary study carried out on the dose response effect of *Murraya koenigii* leaf extract against doxorubicin-induced cardiotoxicity in Wistar rats. Healthy male and female Wistar albino rats were randomly divided into five groups of ten animals in each with equal number of male and female rats in each group. Male and female rats were allocated randomly using a computer-generated randomization table to minimize variation between groups. Group I (control group) received distilled water orally for 14 days, and on the 11^th^ day, single intraperitoneal injection of 10 mL/kg normal saline was administered after a 16 hr fast. In group II (plant extract control), animals were orally administered with 2.0 g/kg lyophilized plant extract for 14 days, and on the 11^th^ day, single intraperitoneal injection of 10 mL/kg normal saline was given after a 16 hr fast. In group III (doxorubicin control group), animals were given distilled water orally for 14 days and a single dose of 18 mg/kg doxorubicin was intraperitoneally injected on the 11^th^ day after a 16 hr fast. Group IV received 2.0 g/kg lyophilized plant extract orally for 14 days, and on the 11^th^ day, single intraperitoneal injection of doxorubicin (18 mg/kg) was administered after a 16 hr fast. Group V was the positive control group and they received distilled water orally for 14 days and a single intraperitoneal injection of dexrazoxane (DZR) (180 mg/kg) was administered 30 min before the administration of single intraperitoneal injection of doxorubicin (18 mg/kg) on the 11^th^ day after a 16 hr fast. At the end of the experimental period all animals were fasted (16 hrs) and sacrificed by cervical dislocation. Blood samples were collected by cardiac puncture for the assessment of cardiotoxicity using cardiac biomarkers including cardiac troponin I (cTnI) concentration, N terminal-pro brain natriuretic peptide (NT-pro BNP) concentration, aspartate aminotransferase (AST, EC 2.6.1.1) activity, and lactate dehydrogenase (LDH, EC 1.1.1.27) activity [26]. Myeloperoxidase (MPO, EC 1.11.2.2) activity was measured to assess the extent of inflammation [24]. Right half of the heart was collected into phosphate buffered saline (PBS) for the estimation of oxidative stress using antioxidant parameters including reduced glutathione (GSH), glutathione peroxidase (GPx, EC 1.11.1.9), glutathione reductase (GR, EC 1.8.1.7), catalase (EC 1.11.1.6) activity, superoxide dismutase (SOD, EC 1.15.1.1) activity, total antioxidant level, and lipid peroxidation [24, 27]. Left half of the heart was collected into 10% formal saline for the histological assessment of cardiac damage as it is the gold standard to detect cardiotoxicity [4].

### 2.5. Assessment of Blood Parameters

The collected blood was centrifuged and serum was separated. cTnI concentration and NT-pro BNP concentration were estimated using commercially available enzyme-linked immunosorbent assay (ELISA) kits purchased from Elabscience Biotechnology Co., Ltd, China. AST activity was measured by using a commercially available colourimetric enzyme assay kit purchased from Biorex Diagnostic, United Kingdom. Commercially available spectrophotometric enzyme assay kit purchased from Biorex Diagnostic, United Kingdom, was used to measure LDH activity in serum. MPO activity was estimated by using commercially available ELISA kit purchased from DRG International Inc., United States of America (USA).

### 2.6. Assessment of Antioxidant Parameters and Lipid Peroxidation in Homogenate of Heart Tissues

The right halves of the rat hearts were collected into ice-cold PBS buffer (pH 7.4) and they were used to prepare the homogenate. Heart tissue was weighed into a homogenizer tube and ice-cold PBS buffer was added (the ratio of tissue weight to homogenization buffer was 1 : 10) to homogenize the heart tissue. The supernatant was collected into a prechilled fresh microcentrifuge tube to assess antioxidant parameters and lipid peroxidation. Total antioxidant level, GSH level, and GR and GPx activities were estimated using commercially available spectrophotometric assay kits purchased from Biorex Diagnostic, United Kingdom. Catalase activity was measured using a commercially available test kit purchased from antibodies-online.com, USA. SOD activity and lipid peroxidation were estimated using commercially available colourimetric assay kits purchased from Sigma Aldrich, USA.

### 2.7. Histological Assessment of Cardiac Damage

Myocardial tissue was sampled from the left half of the heart of all animals for histological assessment and fixed in 10% formal saline. They were processed, sectioned in 3 *µ*m thickness, and stained with haematoxylin and eosin. The sections were examined under the light microscope and necrotic changes were scored. Scoring of necrotic changes was performed according to a grading system developed by the authors as follows: 0, no cells with necrotic changes; 1, up to 10 cells with necrotic changes; 2, 10–50 cells with necrotic changes; 3, 50–100 cells with necrotic changes; 4, >100 cells with necrotic changes. Myocytes with nuclear pyknosis or karrheorhexis or karyolysis with hypereosinophilic cytoplasm and no striation were identified as necrotic myocytes. Necrotic myocyte density was assessed separately in peripheral and subendocardial regions of the myocardium.

### 2.8. Statistical Analysis

Data were expressed as the mean ± standard deviation (SD). One-way analysis of variance followed by Dunnett's multiple comparisons test was used to analyse the statistical difference between different treatment groups using SPSS 22.0 software. Differences were considered statistically significant at *p* < 0.05.

## 3. Results

### 3.1. Acute and Subchronic Toxicity Evaluation of Plant Extract

In acute toxicity study, neither mortality nor morbidity was observed in rats throughout the 14-day period following the single oral administration of the *Murraya koenigi* (L.) Spreng. leaf extract. Morphological characteristics (fur, skin, eyes, and nose) also appeared normal. No tremors, convulsions, salivation, diarrhea, lethargy, or unusual behavior was observed.

**Figure 1:**
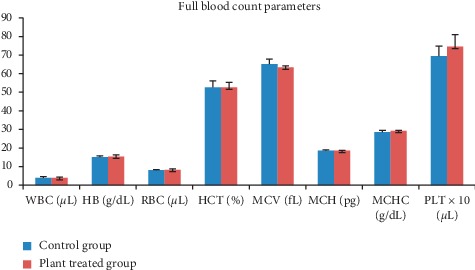
Effect of subchronic administration of *Murraya koenigii* (L.) Spreng. plant extract on haematological parameters. Each column represents the mean ± SD (*n* = 10). ^*∗*^*p* < 0.05 compared to the control. Control group: distilled water orally for 30 days; plant-treated group: aqueous *Murraya koenigii* (L.) Spreng. lyophilized leaf extract orally for 30 days. WBC: white blood cell count, HB: hemoglobin concentration; RBC: red blood cell count; HCT: haematocrit; MCV: mean corpuscular cell volume; MCH: mean corpuscular hemoglobin; MCHC: mean corpuscular hemoglobin concentration; PLT: platelet count.

**Figure 2:**
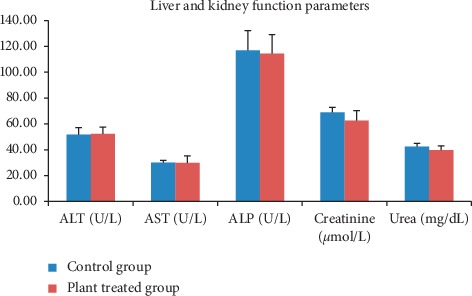
Effect of subchronic administration of *Murraya koenigii* (L.) Spreng. plant extract on liver and kidney function parameters. Each column represents the mean ± SD (*n* = 10). ^*∗*^*p* < 0.05 compared to the control. Control group: distilled water orally for 30 days; plant-treated group: aqueous *Murraya koenigii* (L.) Spreng. lyophilized leaf extract orally for 30 days. ALT: alanine aminotransferase; AST: aspartate aminotransferase; ALP: alkaline phosphatase.

**Figure 3:**
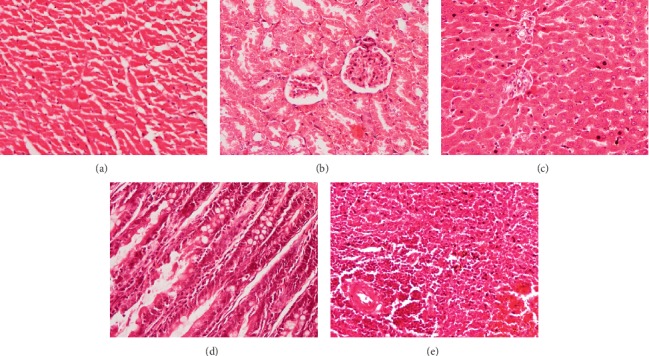
Photomicrographs of haematoxylin and eosin stained tissues of control group of rats which received distilled water orally for 30 days. Magnification (×400). (a) Heart, (b) kidney, (c) liver, (d) small intestine, and (e) spleen.

**Figure 4:**
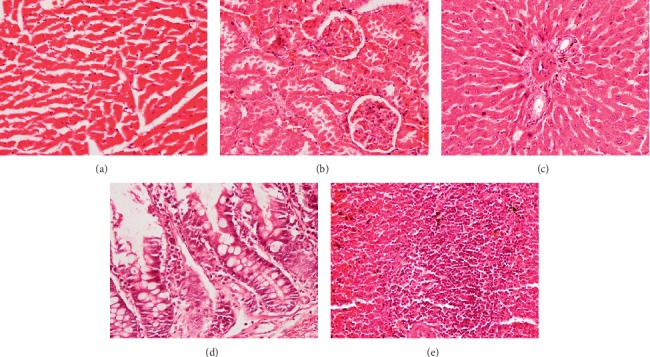
Photomicrographs of haematoxylin and eosin stained tissues of plant extract treated rats which received *Murraya koenigii* (L.) Spreng. lyophilized leaf extract (2.0 g/kg) orally for 30 days. Magnification (×400). (a) Heart, (b) kidney, (c) liver, (d) small intestine, and (e) spleen.

In subchronic toxicity study, treatment-related mortality was not observed in rats treated with plant extract. The effect of subchronic administration of *Murraya koenigi* (L.) Spreng. leaf extract on haematological parameters is presented in Figure 1. All the haematological parameters remained within the physiological range throughout the 30-day experimental period and there was no significant difference (*p* > 0.05) between control group and the plant-treated group. Biochemical parameters of the liver and kidney functions of the treated and control rat groups are shown in Figure 2. A subchronic oral administration of aqueous plant extract did not cause significant changes (*p* > 0.05) in serum concentrations of AST, ALT, ALP, creatinine, and blood urea compared to the control group. There was no evidence of histological lesions observed in any of the organs studied in the control group and the plant-treated rat group (Figures 3 and 4).

### 3.2. Cardioprotective Effect of the Plant Extract

#### 3.2.1. Cardiac Biomarkers in Serum

**Figure 5:**
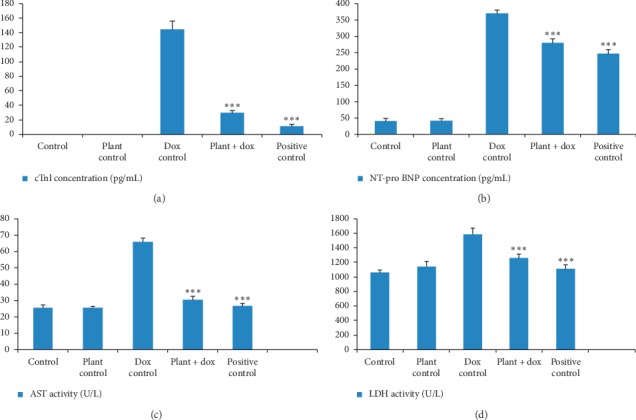
Estimation of cardiac biomarkers in serum. Each column represents the mean ± SD (*n* = 10). ^*∗∗∗*^*p* < 0.001 compared to the doxorubicin control. Control: 10 mL/kg distilled water for 14 days; plant control: 2.0 g/kg lyophilized plant extract for 14 days; dox control: 18 mg/kg doxorubicin on 11^th^ day; plant + dox; 2.0 g/kg lyophilized plant extract for 14 days, 18 mg/kg doxorubicin on 11^th^ day; positive control: 10 mL/kg d H_2_O for 14 days, 180 mg/kg dexrazoxane, 0.5 h before 18 mg/kg doxorubicin on 11^th^ day. (a) cTnI concentration (pg/mL). (b) NT-pro BNP concentration (pg/mL). (c) AST activity (U/L). (d) LDH activity (U/L). cTnI: cardiac troponin I; NT-pro BNP: N-terminal-pro brain natriuretic peptide; AST: aspartate aminotransferase; LDH: lactate dehydrogenase.

The mean concentration of cTnI in serum of rats treated with doxorubicin showed a significantly (*p* < 0.001) increased value (145.15 ± 10.77 pg/mL) compared to the control group which showed zero value for the cTnI concentration as shown in Figure 5. Pretreatment with *Murraya koenigii* (L.) Spreng. leaf extract showed a significant reduction (*p* < 0.001) in serum cTnI concentration with a value of 29.77 ± 2.87 pg/mL compared to the doxorubicin control group. Treatment with plant extract alone did not show any significant change (*p* > 0.05) compared to the control group.

The concentration of NT-pro BNP which is considered as a specific marker in the left ventricular dysfunction was significantly increased (*p* < 0.001) with the value of 371.14 ± 9.69 pg/mL in rats treated with doxorubicin alone compared to the control group (41.57 ± 7.29 pg/mL) as shown in Figure 5. However, the pretreatment with aqueous *Murraya koenigii* (L.) Spreng. leaf extract showed significantly lower (*p* < 0.001) value (218.86 ± 7.91 pg/mL) compared to the doxorubicin treated rats.

The treatment with freeze-dried aqueous plant extract alone did not show any significant difference (*p* > 0.05) in serum AST and LDH levels compared to the control group as shown in Figure 5. The levels of serum AST and LDH levels as markers of heart damage were significantly increased (*p* < 0.001) in doxorubicin-treated rats with respective values of 66.09 ± 2.07 U/L and 1584.19 ± 83.40 U/L compared to the control group which showed respective values of 25.71 ± 1.41 U/L and 1057.21 ± 38.60 U/L. Pretreatment with aqueous leaf extract showed significant reduction (p < 0.001) in these two serum parameters compared to the doxorubicin control group with respective values of 30.52 ± 1.94 U/L and 1256.34 ± 58.20 U/L.

#### 3.2.2. MPO Level in Serum

**Figure 6:**
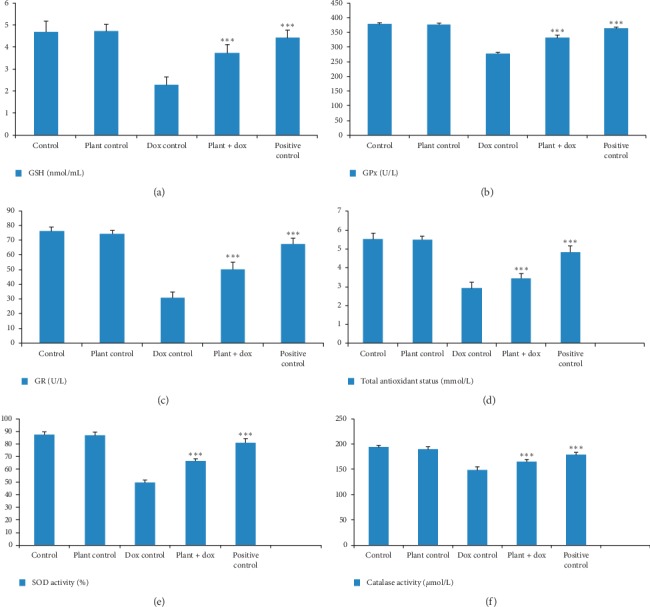
Estimation of oxidative stress markers in homogenate of the heart tissues. Each column represents the mean ± SD (*n* = 10). ^*∗∗∗*^*p* < 0.001 compared to the doxorubicin control. Control: 10 mL/kg distilled water for 14 days; plant control: 2.0 g/kg lyophilized plant extract for 14 days; dox control: 18 mg/kg doxorubicin on 11^th^ day; plant + dox: 2.0 g/kg lyophilized plant extract for 14 days, 18 mg/kg doxorubicin on 11^th^ day; positive control: 10 mL/kg d H_2_O for 14 days, 180 mg/kg dexrazoxane, 0.5 h before 18 mg/kg doxorubicin on 11^th^ day. GSH: reduced glutathione; GPx: glutathione peroxidase; GR: glutathione reductase; SOD: superoxide dismutase. (a) GSH (nmol/mL). (b) GPx (U/L). (c) GR (U/L). (d) Total antioxidant total (mmol/L). (e) SOD activity (%). (f) Catalase activity (*µ*mol/L).

MPO is an enzyme elevated in serum especially in inflammatory conditions. The concentration of MPO in serum of five groups of rats in the present study is shown in Figure 6. The treatment with *Murraya koenigii* (L.) Spreng. plant extract alone did not show any significant difference (*p* > 0.05) compared to the control group. The group of rats treated with doxorubicin alone showed significant increase (*p* < 0.001) in MPO concentration (285.32 ± 1.64 AAU/mL) compared to the control group (157.74 ± 1.76 AAU/mL) and pretreatment with lyophilized plant extract was effective in significantly decreasing (*p* < 0.001) the MPO concentration in serum compared to the doxorubicin control group.

#### 3.2.3. Antioxidant Parameters in Homogenate of Heart Tissues

**Figure 7:**
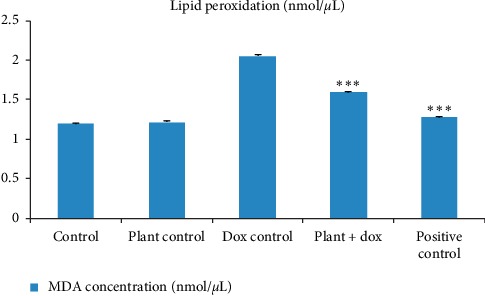
Estimation of MDA concentration in homogenate of the heart tissues as a marker of lipid peroxidation. Each column represents the mean ± SD (*n* = 10). *∗∗∗p* < 0.001 compared to the doxorubicin control. Control: 10 mL/kg distilled water for 14 days; plant control: 2.0 g/kg lyophilized plant extract for 14 days; dox control: 18 mg/kg doxorubicin on 11^th^ day; plant + dox: 2.0 g/kg lyophilized plant extract for 14 days, 18 mg/kg doxorubicin on 11^th^ day; positive control: 10 mL/kg d H_2_O for 14 days, 180 mg/kg dexrazoxane, 0.5 h before 18 mg/kg doxorubicin on 11^th^ day.

Figure 7 shows levels of GSH, GPx, and GR in five different groups of rats used in the study. GSH is an antioxidant which is capable of preventing damage to important cellular components caused by ROS such as free radicals, peroxides, and heavy metals. Myocardial tissues of rats treated with doxorubicin alone in the current study showed significant reduction (*p* < 0.001) in GSH level (2.30 ± 0.37 nmol/mL) compared to the control group (4.70 ± 0.49 nmol/mL). However, the treatment with lyophilized *Murraya koenigii* (L.) Spreng. leaf extract had the ability to significantly increase (*p* < 0.001) the GSH level up to 3.72 ± 0.37 nmol/mL in heart tissues compared to the doxorubicin control group. GPx and GR are important antioxidant enzymes participating in the glutathione-ascorbate cycle. Treatment with *Murraya koenigii* (L.) Spreng. leaf extract alone did not show any significant difference (*p* > 0.05) in these enzymes activities compared to the control. Treatment with doxorubicin in Wistar rats showed a marked reduction (*p* < 0.001) of these two enzyme levels exhibiting the values of 277.60 ± 6.03 U/L and 30.84 ± 4.15 U/L, respectively, in the myocardial tissues compared to the control group which showed 378.25 ± 3.81 U/L and 76.04 ± 3.09 U/L, respectively. Cotreatment with *Murraya* leaf extract showed a significant increase (*p* < 0.001) in these two enzyme levels where 331.10 ± 8.22 U/L for GPx activity and 50.28 ± 4.88 U/L for GR activity were observed.

SOD and catalase are also well-known antioxidant markers in the heart tissues and their mean values in the present study are shown in Figure 7. No significant changes (*p* > 0.05) were observed in these two parameters in the heart tissues of rats treated with *Murraya koenigii* (L.) Spreng. leaf extract alone compared to the control. Doxorubicin treatment in Wistar rats caused a significant decrease in SOD and catalase activities in the myocardial tissues compared to the control. Pretreatment with lyophilized aqueous extract of *Murraya koenigii* (L.) Spreng. leaves could significantly increase (*p* < 0.001) these two enzyme levels in the heart tissues compared to the doxorubicin control.

Total antioxidant status of the heart tissues of five groups of rats in the present study is shown in Figure 7 and rat group treated with doxorubicin alone showed a significant reduction (*p* < 0.001) in the total antioxidant status (2.92 ± 0.32 mmol/L) compared to the control group of rats (5.52 ± 0.33 mmol/L). Pretreatment with the plant extract showed a significant increase (*p* < 0.001) in total antioxidant status with the value of 3.45 ± 0.27 mmol/L compared to the doxorubicin control group.

#### 3.2.4. Lipid Peroxidation Level in Homogenate of Heart Tissues

**Figure 8:**
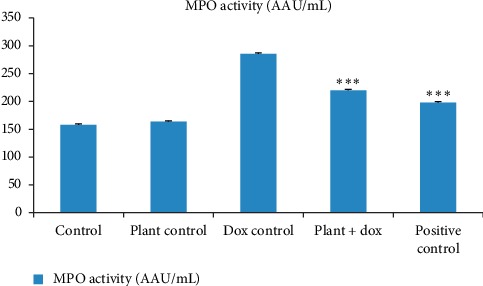
Estimation of MPO activity in serum as a marker of inflammation. Each column represents the mean ± SD (*n* = 10). ^*∗∗∗*^*p* < 0.001 compared to the doxorubicin control. Control: 10 mL/kg distilled water for 14 days; plant control: 2.0 g/kg lyophilized plant extract for 14 days; dox control: 18 mg/kg doxorubicin on 11^th^ day; plant + dox: 2.0 g/kg lyophilized plant extract for 14 days, 18 mg/kg doxorubicin on 11^th^ day; positive control: 10 mL/kg d H_2_O for 14 days, 180 mg/kg dexrazoxane, 0.5 h before 18 mg/kg doxorubicin on 11^th^ day. MPO: myeloperoxidase.

Malondialdehyde (MDA) concentration in myocardial tissues of five groups of rats used in the current study was measured as the end product of lipid peroxidation, and results are presented in Figure 8. Treatment with the lyophilized plant extract did not show any significant difference (*p* > 0.05) in the MDA concentration compared to the control. Doxorubicin treatment in Wistar rats caused a significant increase (*p* < 0.001) in lipid peroxidation with the evidence of increased MDA concentration (2.05 ± 0.02 nmol/*µ*L) compared to the rats in the control group (1.19 ± 0.01 nmol/*µ*L). Pretreatment with *Murraya koenigii* (L.) Spreng. leaf extract showed a significant reduction (*p* < 0.001) of the MDA concentration (1.60 ± 0.01 nmol/*µ*L) compared to the doxorubicin control group.

Dexrazoxane was used as the positive control as it is the only protective agent used in the clinical setting to treat doxorubicin-induced cardiotoxicity. In terms of the biochemical results of the current study, there was a significant difference (*p* < 0.001) between the dexrazoxane-treated group (positive control) and the doxorubicin control. Although the antioxidant parameters and diagnostic markers of myocardial damage showed a significant difference (*p* < 0.05) between the positive control and the plant extract pretreated group, pretreatment with *Murraya koenigii* (L.) Spreng. leaf extract showed a significant difference (*p* < 0.001) in all parameters measured in this study against the doxorubicin control group and showed a remarkable protection against doxorubicin-induced cardiotoxicity in Wistar rats.

#### 3.2.5. Histology of the Myocardial Tissues

**Figure 9:**
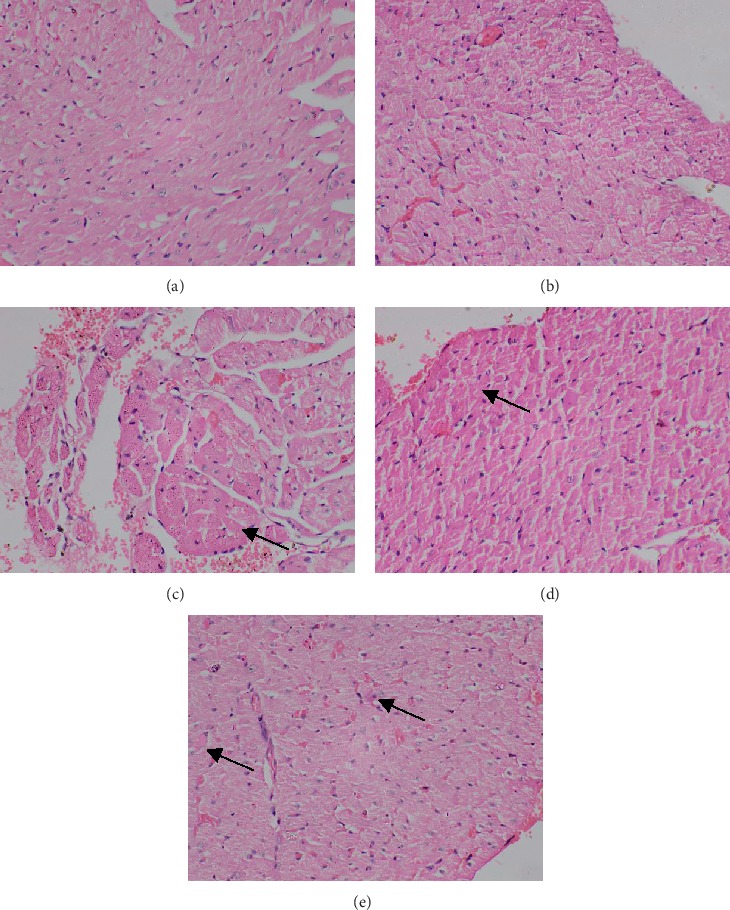
Photomicrographs of subendocardial region of rat myocardium (H & E, ×400). (a) Normal control showing normal morphology, (b) plant extract control showing normal morphology, (c) doxorubicin control showing a large area with early changes of necrosis (myocytes with hypereosinophilic cytoplasm and nuclear changes in cell death: pyknosis, karrheorhexis, and karyolysis), (d) from rats treated with Murraya koenigii leaf extract + doxorubicin showing a smaller area of early changes of necrosis, and (e) positive control group (dexrazoxane + doxorubicin) showing occasional cells with early changes of necrosis. Arrows in each photograph indicate areas/cells of subendocardium with early changes of necrosis.

**Figure 10:**
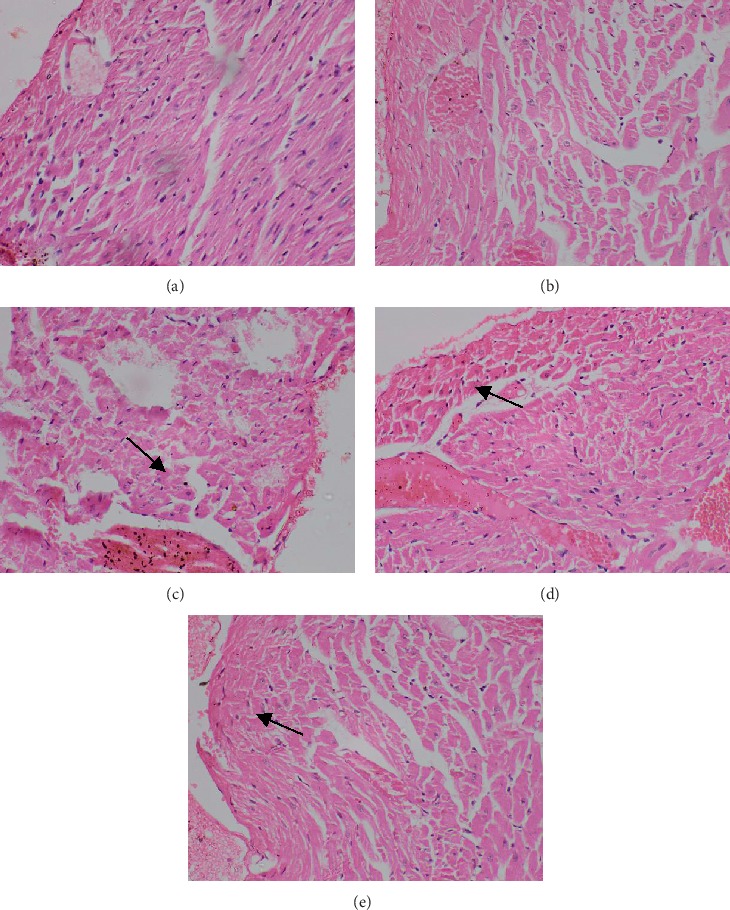
Photomicrographs of peripheral region of rat myocardium (H & E, ×400). (a) Normal control with normal morphology, (b) plant extract control with normal morphology, (c) doxorubicin control showing a large area with early changes of necrosis, (d) from rats treated with *Murraya koenigii* leaf extract + doxorubicin showing a smaller area of early changes of necrosis, and (e) positive control group (dexrazoxane + doxorubicin) showing occasional cells with early changes of necrosis. Arrows in each photograph indicate areas of peripheral region of myocardium with early changes of necrosis. Necrosis is less pronounced in the peripheral region of the myocardium compared to the subendocardial region.

**Figure 11:**
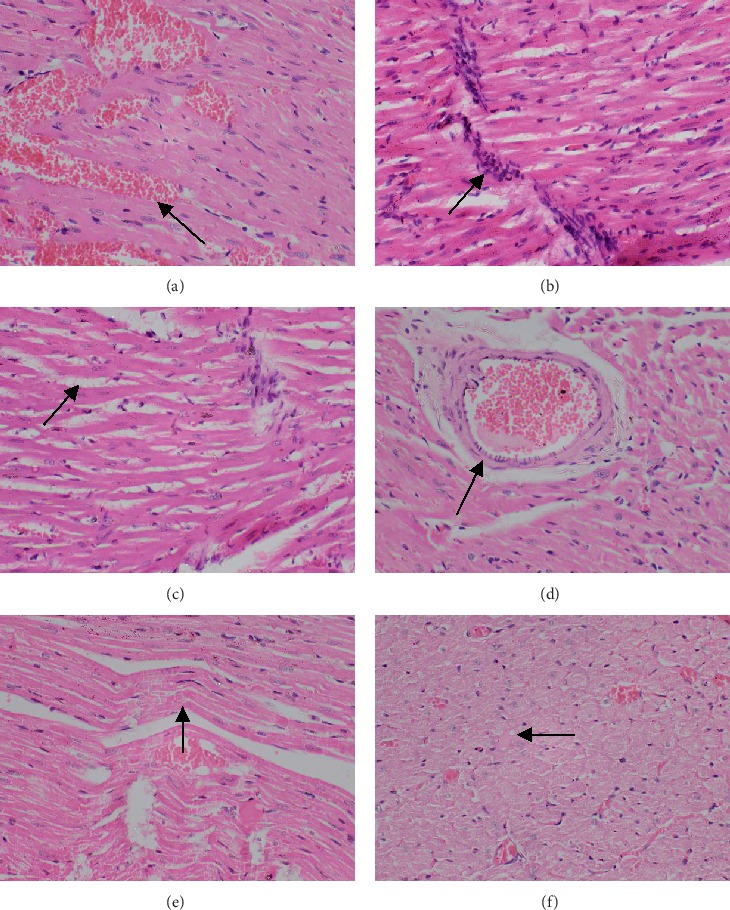
Photomicrographs of reversible histological changes in myocardium of rats (H & E, ×400): (a) haemorrhages, (b) inflammatory infiltrations, (c) interstitial oedema, (d) congestion of blood vessel, (e) wavy myocardial fibers, and (f) intracellular vacuoles. Arrows in each photograph indicate reversible histological changes.

Transverse sections of the myocardial tissues of the control group showed normal myocardial architecture in both subendocardial and peripheral region of the myocardium (Figures 9 and 10). Large areas with early cellular changes of necrosis including pyknotic nuclei and hypereosinophilic cytoplasm were seen in the myocardium of the rats treated only with doxorubicin and showed a total score of 7.8 (Table 1). Necrotic areas were more visible in the subendocardial region compared to the peripheral region of the myocardial tissues (Figures 9 and 10). Reversible changes of cell injury, including haemorrhages, interstitial oedema, inflammatory cell infiltration, congestion of blood vessels, wavy myocardial fibers, and intracellular vacuoles (Table 2, Figure 11), were seen in rats treated only with doxorubicin. However, pretreatment with the aqueous extract of *Murraya koenigii* (L.) Spreng. leaves showed a smaller area with early changes of necrosis with a mean score of 4.2 (Table 1) and necrotic areas were more pronounced in the subendocardial region of the myocardial tissues. This group of rats showed lesser degree of cellular changes of necrosis compared to the doxorubicin control group (Figure 9). Occasional wavy myocardial fibers, intracellular vacuoles, and congestion of blood vessels were observed as reversible histological changes while haemorrhages, inflammatory cell infiltrations, and interstitial oedema were absent in this group of animals (Table 2). Treatment with *Murraya koenigii* (L.) Spreng. leaf extract alone did not show histological evidence of reversible or irreversible cell injury and showed normal morphology of the myocardial tissues as in the control group (Figures 9 and 10). Taking into consideration the histology of the positive control group, they showed better preserved myocardium by giving only 2.7 of the total score for early changes of necrosis and only intracellular vacuoles were observed as histological evidence of reversible cell injury.

## 4. Discussion

Anthracyclines, including doxorubicin, epirubicin, daunourubicin, and idarubicin, play a major role in chemotherapy and are used to treat many solid organ tumours and haematologic malignancies such as breast, ovarian, lung, uterine, and bladder cancers, and different types of leukaemias and lymphomas [28, 29]. However, it is widely reported that dose-dependent cardiotoxicity of anthracyclines limit their clinical effectiveness [1–4, 28, 29]. Although it is accepted that high cumulative doses of doxorubicin >400 mg/m^2^ may cause cardiotoxicity in patients, some studies on childhood and adult cancer survivors have shown that cardiomyopathy induced by doxorubicin may occur even with a <400 mg/m^2^ cumulative dose of doxorubicin, and therefore there is no safe dose of anthracycline to be prescribed [6, 30–32]. Although multiple mechanisms are involved in doxorubicin-induced cardiotoxicity, generation of ROS which causes lipid peroxidation and depletion of antioxidant enzymes is considered as the major mechanism involved [32]. Cardiac tissue is more susceptible to doxorubicin-induced cardiotoxicity due to its particular effect on mitochondria [33]. Heart contains a large number of mitochondria as it needs much energy and these mitochondria have high affinity for doxorubicin as their inner membrane contains an anionic phospholipid named cardiolipin which has high binding affinity for the cationic drug, doxorubicin [33, 34]. Enzymes within mitochondria including NADPH dehydrogenase, cytochrome P-450 reductase, and xanthine oxidase have the ability to transform doxorubicin into a semiquinone radical which reacts with molecular oxygen to form superoxide anion which could be further changed to other ROS [33]. Furthermore, endothelial NOS are also responsible for the production of ROS in doxorubicin-induced cardiotoxicity.

As the formation of free radicals is considered as the major culprit in doxorubicin-induced cardiotoxicity, antioxidant compounds have been recognized as potential therapeutic agents [32]. The only synthetic drug prescribed to prevent cardiotoxicity in clinical settings is dexrazoxane but it has several limitations such as interference with antitumour efficacy of doxorubicin, not being approved for the use in children, and increased risk of secondary malignancies [29]. Therefore, replacing the synthetic drugs with antioxidant compounds present in herbal medicines which have less side effects is considered as a better option [32]. *Murraya koenigii* or curry leaf which is known as karapincha in “Sinhala” is commonly used as a medicinal plant in traditional system of ayurveda in Sri Lanka [35]. *Murraya koenigii* leaves contain phytochemicals including polyphenols, alkaloids, tannins, flavonoids, and reducing sugars and they are rich in polyphenols and flavonoids which have a strong antioxidant potential [9]. Therefore, it was suggested that *Murraya koenigii* (L.) Spreng. leaf extract may have the potential to attenuate doxorubicin-induced cardiotoxicity which may arise as a result of the damage caused by free radical formation. Although the antioxidative, cytotoxic, hypoglycaemic, antidiabetic, antimicrobial, antibacterial, antiulcer, positive inotropic, and cholesterol-reducing activities of *Murraya koenigii* have been reported *in vivo*, its effects against doxorubicin-induced cardiotoxicity has never been investigated [35–41]. Therefore, the current study was conducted to investigate the cardioprotective potential of aqueous extract of *Murraya koenigii* (L.) Spreng. leaves against doxorubicin-induced cardiotoxicity in Wistar rats. A dose response curve was drawn and the best dose of the plant extract selected against doxorubicin-induced cardiotoxicity in Wistar rats was 2.0 g/kg. Acute and subchronic toxicity study for the respective dose of *Murraya koenigii* (L.) Spreng. leaf extract was performed and the results indicated that the selected dose was free of any toxic effects and safe to be used in Wistar rats.

Doxorubicin-induced cardiotoxicity and cardioprotective potential of *Murraya koenigii* (L.) Spreng. leaf extract were investigated by using serum biomarkers of myocardial damage, antioxidant parameters, myeloperoxidase activity, and histological assessment. Concentration of cTnI, NT-pro BNP, AST, and LDH activities were measured as serum biomarkers of myocardial injury. cTnI is considered as one of the most sensitive and commonly used biomarkers, and its release from the cardiomyocytes is proportional to the size and extent of cardiac tissue injury [42]. In the present study, doxorubicin control group showed a marked increase in cTnI concentration indicating that there is an extensive damage to the cardiac tissues in the presence of doxorubicin. However, the treatment with *Murraya* leaf extract showed a marked reduction of the cTnI value suggesting that this plant extract has the ability to reduce cardiac cell injury caused by doxorubicin. These results were consistent with the previous studies done by Atas et al. and Shaker et al. [42, 43].

NT-pro BNP is rapidly produced and secreted by the heart in the event of atrial and ventricular distention as in congestive heart failure, and it is also considered as a useful marker of left ventricular dysfunction in patients on anthracycline chemotherapy [44]. Studies done by Argun et al. and Ridha et al. in experimental rats have shown that doxorubicin increases the concentration of serum NT-pro BNP [44, 45]. Similar to these results, the present study showed that doxorubicin treatment alone significantly increases (*p* < 0.001) the NT-pro BNP concentration in the serum compared to the control rats and pretreatment with *Murraya koenigii* (L.) Spreng. leaf extract markedly reduced the elevated NT-pro BNP concentration by showing the cardioprotective effect of the plant extract.

Serum biomarkers of myocardial toxicity including AST and LDH indicate the loss of cell membrane integrity of myocytes [46]. It is believed that enhanced production of free radicals in doxorubicin metabolism may enhance myocyte degeneration and hence increase the enzyme leakage [47]. Although these two are not very specific biomarkers for the detection of myocardial cell injury, their elevation together with more specific and sensitive biomarkers such as cTnI specify the cardiac damage. According to the results of the present study, elevated AST and LDH levels were significantly reduced by the pretreatment with aqueous plant extract compared to the doxorubicin control. Many previous studies have also shown that compounds with antioxidant property may bring down the cardiac biomarkers in rats treated with doxorubicin [47–49].

Antioxidant compounds and antioxidant enzymes including GSH, GPx, GR, SOD, and catalase protect the body against oxidative-stress-mediated tissue injury [47]. GSH is considered as the most important intracellular hydrophilic antioxidant which protects the cells from free radical damage [50]. As doxorubicin causes excessive formation of superoxide radicals, it results in the excessive formation of hydrogen peroxide [51]. This hydrogen peroxide is neutralized by GPx using hydrogen from GSH molecules which produces water and oxidized glutathione [50]. Regeneration of GSH occurs in the presence of GR by reducing oxidized glutathione. Therefore, doxorubicin treatment which causes excessive production of superoxide radicals significantly reduced (*p* < 0.001) the level of GSH, GPx, and GR in Wistar rats. However, pretreatment with *Murraya koenigii* (L.) Spreng. leaf extract significantly increased the GSH, GPx, and GR level in Wistar rats suggesting that antioxidant property of this plant extract may restore the cardiomyocytes with antioxidants. Consistent to the results that we reported, Asfar et al. also reported that doxorubicin treatment decreased the activities of antioxidant enzymes and *Acacia hydaspica* leaf extract that has the potential to increase GSH, GPx, and GR level which was attributed by polyphenolic constituents and antioxidant properties of this leaf extract [50]. A study done by Chen et al. has shown that diosgenin, a steroidal saponin of *Dioscorea opposita*, also has the ability to increase the GSH and GPx level decreased by doxorubicin treatment in Balb/c mice [48]. Thymoquinone, an active constituent of *Nigella sativa*, having antioxidant and anti-inflammatory actions was also shown to protect against doxorubicin-induced cardiotoxicity in mice with increased value of antioxidant markers including GSH, GPx, and GR activities [52].

SOD, catalase, and GPx are three key enzymes considered as the first line defense antioxidant enzymes which dismutate superoxide radical and breakdown hydrogen peroxides and hydroperoxide to harmless molecules such as H_2_O_2_/alcohol and O_2_ [53]. Inside the cardiomyocytes, doxorubicin undergoes redox cycling and produces semiquinone radical which is oxidized back to its original form by reducing molecular oxygen to form superoxide radicals [4]. SOD is a metaloenzyme which catalyzes the conversion reaction of superoxide radicals to hydrogen peroxide [52]. This hydrogen peroxide is converted to water and molecular oxygen by catalase enzyme. Therefore, excessive ROS formation in doxorubicin treatment decreases the ability of cardiomyocytes to detoxify ROS as it decreases the level of SOD and catalase. However, the treatment with aqueous *Murraya* leaf extract increases SOD and catalase level suggesting its antioxidant and ROS-scavenging nature. Not only the present study but also many previous studies on the protective effect against doxorubicin-induced cardiotoxicity based on the antioxidant property have shown a similar effect on SOD and the catalase activity [47, 48, 51, 52, 54]. Total antioxidant status of the doxorubicin-treated animals was greatly reduced in the present study suggesting that doxorubicin treatment causes depletion of antioxidants in the body. Pretreatment with aqueous leaf extract showed increased level of total antioxidant status confirming the antioxidant activity of *Murraya* leaf extract. Results of this present study were in harmony with previous studies done by Al-Harthi et al. and Hamza et al. [51, 55].

The superoxide anions and their derivatives specially the highly reactive and damaging hydroxyl radicals formed in doxorubicin metabolism cause the lipid peroxidation of the cell membrane [51]. A significant increase in lipid peroxidation was observed in the current study as evidenced by increased MDA level in the homogenate of the heart tissues of rats treated with doxorubicin alone. MDA is a stable end product of lipid peroxidation and is regarded as a reliable marker for estimation of free radical-induced tissue damage and lipid peroxidation [56]. Pretreatment with *Murraya koenigii* (L.) Spreng. leaf extract significantly reduced the lipid peroxidation suggesting the antioxidant potential of the plant. Many previous studies on natural products or other compounds with high amount of antioxidants have shown a reduction of lipid peroxidation in doxorubicin-induced cardiotoxicity [27, 51, 52, 54, 55].

Increased oxidative stress in doxorubicin-induced cardiotoxicity may lead to expression of nuclear factor kappa B (NF-*κ*B). Activation of nucleotide-binding oligomerization domain- (NOD-) like receptor protein 3 (NLRP3) increases release of proinflammtory cytokines in the myocardium such as tumour necrosis factor alpha (TNF-*α*) and interleukin-1 beta (IL-1*β*). These proinflammatory cytokines concurrently may increase the MPO activity [55]. Therefore, measurement of MPO activity in serum is considered as a reliable marker to identify inflammation in doxorubicin-induced cardiotoxicity. In the current study, MPO activity in doxorubicin-treated rats was elevated confirming that oxidative stress induced by doxorubicin may initiate inflammation. However, pretreatment with *Murraya* leaf extract reduced the MPO activity proving its antioxidant and anti-inflammatory activities. Studies done by Hamza et al. and Sun et al. have also shown similar results as in this present study [24, 55, 57].

Observation of histological changes in myocardial tissue is considered as the gold standard to detect acute doxorubicin-induced cardiotoxicity [4]. A study done by Hamza et al. showed that doxorubicin-treated rats showed extensive tissue damage with multiple necrotic foci of myocardial fibers with cytoplasmic vacuole formation and induced eosinophilia, marked oedema, and accumulation of inflammatory cells. Pretreatment with a high dose of *Melissa officinalis* showed a well-preserved appearance of myocardial fibers with slight degree of oedema [55]. These results were very much consistent with the results of the present study. Another study done by Shaker et al. also reported that Wistar rats treated with 15 mg/kg cumulative dose of doxorubicin showed histological changes including congestion of myocardial vessels, myocardial swelling, myocardial fibers disorganization, cytoplasmic vacuolization, perinuclear vacuolization, and myofibrillar loss, and rats treated with enoxaparin showed mild to moderate score in tissue damage and displayed less cytoplasmic or perinuclear vacuolization and no myofibrillar loss [42]. These results were also in line with the present study. Not only these studies but also many other previous studies were consistent with the results of the present study [16, 24, 54]. In the present study, doxorubicin-treated rats showed large areas with early changes of necrosis and reversible histological changes including inflammatory infiltrations, interstitial oedema, haemmorrhage, congestion of blood vessels, wavy myocardial fibers, and intracellular vacuoles. Pretreatment with aqueous *Murraya koenigii* (L.) Spreng. leaf extract showed lesser degree of cardiac damage with smaller area of early changes of necrosis and intracellular vacuoles, congestion of blood vessels, and wavy myocardial fibers as reversible histological changes in myocardial tissues indicating cardioprotective potential of the leaf extract.

According to the overall biochemical and histological results of the present study, it was clearly observed that aqueous extract of *Murraya koenigii* (L.) Spreng. leaves had the potential to significantly reverse the increased values of cardiac biomarkers of myocardial injury, oxidative stress markers, inflammatory marker, and reversible histological changes of the myocardium. Further, *Murraya* leaf extract had ability to decrease the degree of irreversible histological changes. All these attenuation effects may be attributed by cardioprotective, antioxidative, and anti-inflammatory effects of *Murraya koenigii* (L.) Spreng. leaf extract.

## 5. Conclusion

The overall protective effect of *Murraya koenigii* (L.) Spreng. leaf extract may be due to the counter action with free radicals by its antioxidant nature which suggests that pretreatment with *Murraya koenigii* (L.) Spreng. leaf extract may replenish the cardiomyocytes with antioxidants that are needed for the defense against oxidative stress induced by doxorubicin. However, the exact molecular mechanism of *Murraya* leaf extract which exerts its protective action against oxidative damage remains to be investigated. If the protective function will be confirmed in cancer patients, *Murraya koenigii* (L.) Spreng. leaf extract may be used as an adjuvant therapy with doxorubicin.

## Figures and Tables

**Table 1:** Average grading of cells with necrotic changes in myocardial tissues.

	Group I (control)	Group II (plant extract control)	Group III (dox control)	Group IV (plant + dox)	Group V (positive control)
Subendocardial region of heart tissues (score out of 4)	0	0	4	2.9	1.9
Peripheral region of heart tissues (score out of 4)	0	0	3.8	1.3	0.8
Total score (out of 8)	0	0	7.8	4.2	2.7

**Table 2:** Reversible histological changes in myocardial tissues.

Reversible histopathological changes	Group I (control)	Group II (plant extract control)	Group III (dox control)	Group IV (plant + dox)	Group V (positive control)
Haemorrhages	Absent	Absent	Present	Absent	Absent
Interstitial oedema	Absent	Absent	Present	Absent	Absent
Inflammatory infiltrations	Absent	Absent	Present	Absent	Absent
Intracellular vacuoles	Absent	Absent	Present	Present	Present
Congestion of blood vessels	Absent	Absent	Present	Present	Absent
Wavy myocardial fibers	Absent	Absent	Present	Present	Absent

## Data Availability

The datasets used and/or analyzed during the current study are available from the corresponding author on reasonable request.
